# Clinical evaluation of a novel radiofrequency‐based toothbrush for teeth whitening and reduction of teeth stains: A pilot study

**DOI:** 10.1002/cre2.460

**Published:** 2021-06-17

**Authors:** Shadi Shehadeh, Liora Levi, Dror Shamir

**Affiliations:** ^1^ Oral and Maxillofacial Surgery Unit Bnai Zion Medical Center Haifa Israel; ^2^ Clinical Research Department Home Skinovations Ltd Yokneam Israel

**Keywords:** radiofrequency, teeth stains, teeth whitening, toothbrush

## Abstract

**Objectives:**

To evaluate the effect of a novel radiofrequency (RF)‐utilizing toothbrush on reduction of stains and improvement of teeth shade.

**Materials and Methods:**

This was an open label, prospective study, including six clinical visits that were conducted over a period of 8 weeks. Subjects performed twice daily brushing using a novel RF‐utilizing toothbrush, used manually with no mechanical vibration. Teeth shade and stains were assessed using Vita Easyshade advanced 4.0 device and a visual assessment of standardized digital photographs, before, and following 4 and 8 weeks of treatment.

**Results:**

Twelve subjects completed the study with fully evaluable data. A notable shade improvement (whitening) was observed between the visits according to the average scores of teeth whitening index per subject. Statistical analysis conducted by the unstructured mix model confirmed a significant reduction in the average score of whitening index between baseline and visit 5 (*p* < 0.001) as well as between baseline and visit 6 (*p* < 0.001). No significant difference was found between the average teeth shade in visit 5 as compared to visit 6 (*p* = 0.918). No significant difference was found in teeth shade between females and males; however, subjects age was significantly correlated with teeth shade (*p* < 0.001). Digital photographs indicated a notable reduction in visible dark stains. The toothbrush was well tolerated and no device‐related adverse events were reported during the study.

**Conclusions:**

The novel RF‐utilizing toothbrush produced significant benefits relating to teeth whitening and stains reduction, following one and 8 weeks of brushing.

## 1 INTRODUCTION

Over the past two decades, tooth bleaching or whitening has become one of the most popular esthetic dental treatments (Sulieman, 2008). Current tooth bleaching materials are based primarily on either hydrogen peroxide or carbamide peroxide, both found to be effective, but raise concerns about the long‐term safety of bleaching procedures, referring to the tooth structure, pulp tissues, and the mucosal tissues of the mouth, as well as systemic ingestion (Dahl & Pallesen, 2003; Goldberg et al., 2010; Mahaseth & Kuzminov, 2017; Minoux & Serfaty, 2008; Schallreuter & Elwary, 2007). Recently, products containing chlorine dioxide were introduced, but with additional safety concerns due to the low pH of the material (Greenwall, 2008).

At‐home bleaching methods include gels, chewing gums, rinses, toothpastes, paint‐on films, and whitening strips (Clifton, 2014). Toothpastes and mouth washes which are advertised as “whitening” rarely contain peroxide derivatives in low concentrations (Clifton, 2014). These toothpastes are abrasive (usually containing alumina or silica) for the purpose of removing surface stains from the tooth surface (Clifton, 2014), can cause thinning of the enamel layer, and contributes to a gradual darkening in the appearance of the tooth as the dentin layer becomes more noticeable (Hara & Turssi, 2017).

**FIGURE 1:**
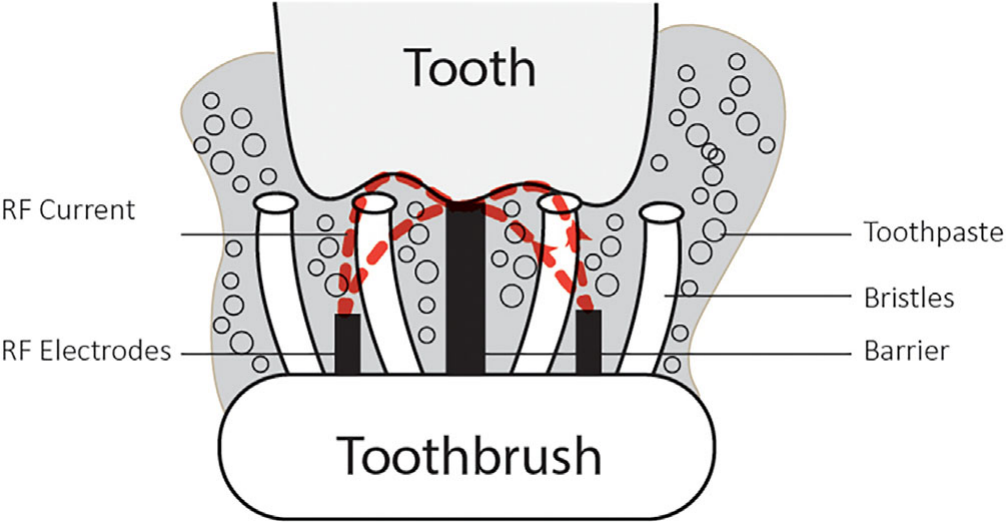
Schematic representation of the radiofrequency current on the tooth surface

With an aim to provide safe and efficient reduction of extrinsic stains at home and teeth whitening, and without changing the daily dental hygiene routine, HomeSkinovations, Ltd. (Yokneam, Israel) has developed a RF‐utilizing toothbrush. It is a novel toothbrush, shown to remove effectively the impurities (i.e., stains, calculus) that are strongly attached to the tooth surface, to significantly improve the dental hygiene and thus to promote the reduction of gingival inflammation (Milleman, Grahovac, et al., 2020; Milleman, Levi, et al., 2020). The RF‐based toothbrush works in a non‐abrasive way; it utilizes low‐power RF energy that streams between two electrodes and over a silicon barrier and reaches the tooth surface during brushing (see Figure 1). RF is an alternating electric current that oscillates at radio frequencies in the range of 3 kHz–300 GHz. It has been used in medicine for several decades for many different applications, from surgical to esthetic, providing various effects, depending on the specific parameters of the device in use (Belenky et al., 2012). Specifically, the novel toothbrush RF technology is proposed to bring charged molecules that originate from the toothpaste, to the tooth surface, in order to destabilize the electrostatic bonds between the tooth and the impurities (calculus, stains, plaque) that are attached to it. This pilot study was aimed to evaluate the RF technology for patient safety and efficacy in relation to extrinsic stains and teeth shade. For this purpose, the mechanical vibration that is common to all power toothbrushes was not implemented in the present study, to enable the demonstration of the RF effect. Our hypothesis is that RF‐utilizing toothbrush provides a positive change in extrinsic stains and teeth shade.

## 2 MATERIALS AND METHODS

An open label, prospective study, was conducted, in order to evaluate the safety and efficiency of the RF‐utilizing toothbrush for removal of extrinsic dental stains and teeth whitening. The protocol and consent form were approved by the Bnai Zion Medical Center Ethical Approval Committee before study initiation, and verbal and written consent were obtained from all subjects.

### 2.1 Participants

Recruitment was conducted using advertisements that were posted on the hospital website; individuals who were interested in study participation contacted the dental clinic coordinator in order to continue in the recruitment process. All screened subjects signed an informed consent form, and received an oral examination and a dental radiography assessment. Extrinsic dental stains and tooth shade of the 12 anterior teeth were evaluated using a visual assessment and the Vita EasyShade advanced 4.0 spectrophotometer device.

Recruited subjects were 25–70 years of age, with dark shade of the front teeth (including the following scores: A3‐A4, B3‐B4, D3‐D4, or C3‐C4) as assessed by the Vita EasyShade device or notable teeth stains on the facial side of their 12 anterior teeth. Exclusion criteria were composed of current or history of oral cavity cancer or oropharyngeal cancer, any active electrical implant anywhere in the body, pregnant or nursing, and any active condition or surgery in the oral cavity within 3 months prior to treatment. Subjects who did not practice daily oral hygiene were also excluded.

### 2.2 Study procedures

Eligible study participants were provided with regular, marketed Colgate advanced clean (0.32% (wt/wt) Sodium Fluoride toothpaste (Colgate‐Palmolive), and the RF‐utilizing toothbrush. Subjects signed informed consent prior to any study related activities, and were given information about the device. Dental examination was conducted, including dental radiography. Baseline data such as demographic characteristics and relevant medical history was collected, and visit schedule was set. Teeth shade was evaluated for 12 anterior teeth (labial surface) using the Vita EasyShade device. Anterior teeth were photographed using a digital standardized camera and the qualitative extent of extrinsic stains was evaluated by a visual assessment of these digital before and after photos.

Participating subjects were instructed to brush at home twice‐daily (morning and evening) with a full brush head of toothpaste for two timed minutes. The toothbrush was used in a manual manner (no mechanical vibration was applied during brushing). The study included 8 weeks of test phase, during which 6 study visits were performed. The subjects received a designated form for documentation of each brushing session. Participants continued to use their assigned study treatment twice‐daily (morning and evening) for the next 8 weeks, recording each brushing in the diary card provided. Participants returned to the study site once a week during the first month, and for the final 6th visit that was conducted after 8 weeks of brushing. Diaries were checked to assess compliance.

### 2.3 Assessments

Clinical efficacy was evaluated at visits 5 and 6, (following 4 and 8 weeks of brushing, respectively), by comparing the teeth shade before and after treatment. Digital photographs of the anterior teeth were taken as well in order to assess visible dark stains.

Teeth shade evaluations were conducted at baseline, visit 5, and 6, using the Vita EasyShade device, which automatically measures the tooth shade according to the VITA Classical A1–D4 Shade Guide. The average shade of each tooth was calculated automatically by the device according to five singular measurements per tooth. For analysis, each of the 16 shade tabs was translated to teeth whitening index and assigned a number from 1 (dark) to 16 (light) according to the Munsell color ranking system, as instructed in the device manual and described previously (Reis et al., 2011). According to this color ranking system, brighter teeth shades correspond to a lower index number and darker teeth shades correspond to a higher index number, as shown in Table 1.

**TABLE 1:** Vita classical A1‐D4 shade guide conversion table

Vita classical shade guide	Whitening index	
B1	1	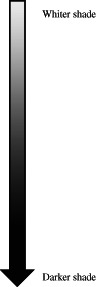
A1	2	
B2	3	
D2	4	
A2	5	
C1	6	
C2	7	
D4	8	
A3	9	
D3	10	
B3	11	
A3.5	12	
B4	13	
C3	14	
A4	15	
C4	16	

### 2.4 Safety

A radiographic and clinical examination was conducted at baseline, in order to exclude dental pathological findings and to ensure eligibility for study procedures. In addition, a thorough evaluation of the oral soft tissues (OST) was conducted at each visit, by way of a visual examination of the oral cavity, including the gingiva (free and attached), hard and soft palate, oropharynx/uvula, buccal mucosa, tongue, floor of the mouth, labial mucosa, mucobuccal/mucolabial folds, lips, and perioral area. A trained dentist performed intraoral examinations at each study visit. In case an adverse event occurred (AE) it was recorded and monitored throughout the study. Any observed abnormalities noted during the OST examination were transcribed beginning at the screening visit until 5 days after the final use of study product. The investigator determined the causal relationship of each AE using his clinical experience and selected the appropriate severity descriptor as mild, moderate, or severe.

### 2.5 Data analysis

A sufficient number of participants were to be screened in order to include 15 subjects. Safety analysis was carried out on a modified intent‐to‐treat (ITT) population, defined as all recruited participants who conducted at least one treatment. Efficacy analysis was conducted on the per‐protocol (PP) population including all participants in the ITT population who had no protocol deviations deemed to affect efficacy.

The reliability of the Vita EasyShade measurements within scales was evaluated using Cronbach's alpha. The average shade of 12 anterior teeth was calculated within each visit per subject. The improvement in teeth shade was evaluated between baseline and 4 weeks as well as between baseline and 8 weeks, using mixed linear model with unstructured covariance matrix accounting for age, gender, race, compliance and average brushing duration.

Significance level was defined as *α* = 0.05. Graphical representation includes means and standard errors. Analyses were carried out using IBM Corp. Released 2020 and IBM SPSS Statistics for Windows, Version 27.0.1 Armonk, NY: IBM Corp.

## 3 RESULTS

A total of 15 subjects provided informed consent and were enrolled to this study, 13 of them met the entrance criteria and were recruited to the study. Out of the 13 completers, one subject was excluded due to protocol deviations, while 12 subjects completed with fully evaluable data at the trial's conclusion. The demographic characteristics of the study population is presented in Table 2. The mean age of the study population was 46.63 years, with a range of 25–68 years; 9 (75.0%) of the subjects were male.

**TABLE 2:** Baseline demographics and characteristics

*N*	12
Age	
Mean ± *SD*	46.63 ± 13.73
Range	25–68
Gender	
Male	9 (75.0%)
Female	3 (25.0%)
Country/continent of origin	
North Africa	4 (33.33%)
Russia	2 (16.67%)
Asia	2 (16.67%)
Europe	2 (16.67%)
Israel	2 (16.67%)

### 3.1 Efficacy

Analysis of Cronbach's alpha was performed in order to evaluate the validity of the results, and confirmed that the study measurements are correlated and reliable (*α* ≥ 0.877).

**FIGURE 2:**
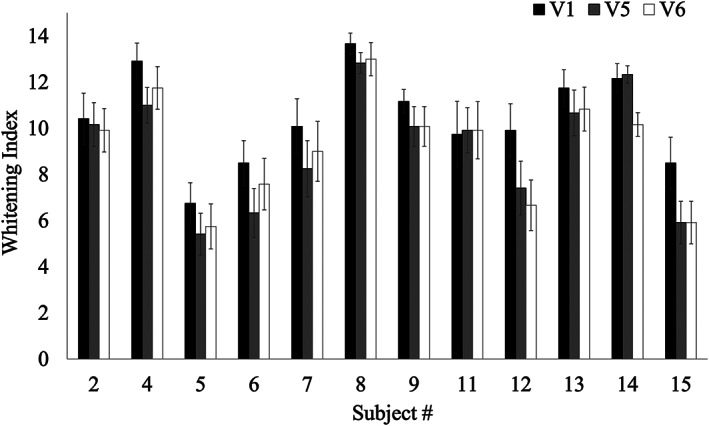
Teeth whitening index per subject, as measured by the Vita EasyShade advanced 4.0 device. Measurements were averaged over 12 anterior teeth. V1, V5, V6 correspond to baseline, 4‐weeks, and 8‐weeks visits

The shade scores (whitening index scores) of individual subjects, as calculated by averaging the scores of 12 anterior teeth at each visit, is shown in Figure 2. The results indicate that most subjects experienced an average reduction in shade index following 4 and 8 weeks of brushing.

**FIGURE 3:**
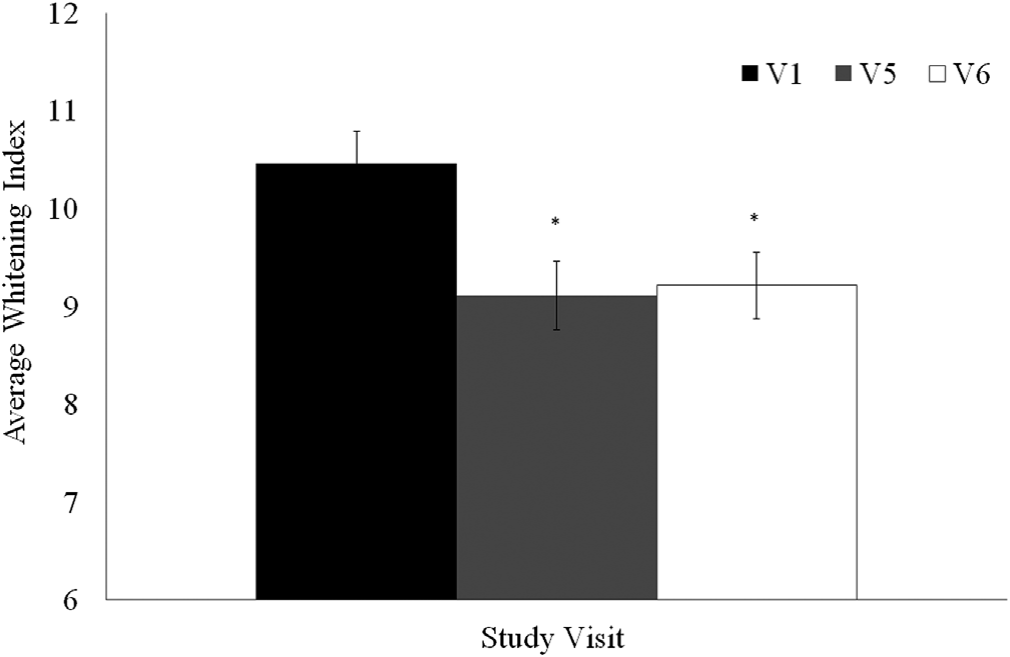
Teeth shade evaluation, as averaged for all 12 subjects before (V1), after 4 (V5) and 8 (V6) weeks of brushing (*p < 0.001)

Figure 3 illustrates the average shade scores, as calculated for 144 teeth of 12 subjects at baseline, visit 5 and visit 6. Statistical analysis confirms a significant reduction in shade scores between baseline and visit 5 (*p* < 0.001) as well as between baseline and visit 6 (*p* < 0.001). The mean scores and 95% CI of mean whitening index scores at BL, 4 and 8 weeks were found to be 10.47 [9.83, 11.10], 9.11 [8.42, 9.81], and 9.22 [8.55, 9.89], respectively. In addition, no significant difference was found between the average teeth shade in visit 5 as compared to visit 6 (*p* = 0.918). Table 3 presents the measured differences in teeth shade scores between the visits, showing differences of 1.25 ± 0.93 and 1.27 ± 0.96 in shade scores between baseline to visit 5 and visit 6 respectively, and a 0.02 difference between visit 5 and visit 6 that is not statistically significant. The effects of age and gender on teeth shade were analyzed as well. Results (shown in Table 3) indicate that there is no significant difference in teeth shade between females and males. According to our analysis, the subjects age significantly influenced teeth shade (*p* < 0.001), as the average whitening index increased by 0.21 points per year, indicating that the teeth shade darkens with age.

**TABLE 3:** Differences in teeth shade average scores in view of various influencing factors

Influencing factor	Difference in teeth shade average score	95% CI delta	*p* Value
Visit 6 vs. BL	−1.250	[−1.821, −0.679]	<0.001
Visit 5 vs. BL	−1.297	[−1.842, −0.752]	<0.001
Visit 5 vs. Visit 6	−0.047	[−0.542, 0.447]	1.000
Female vs. Male	−0.077	[−1.307, 1.153]	0.901
Age	0.206^a^	[0.131, 0.282]	<0.001

^a^ Per age difference of 1 year.

**FIGURE 4:**
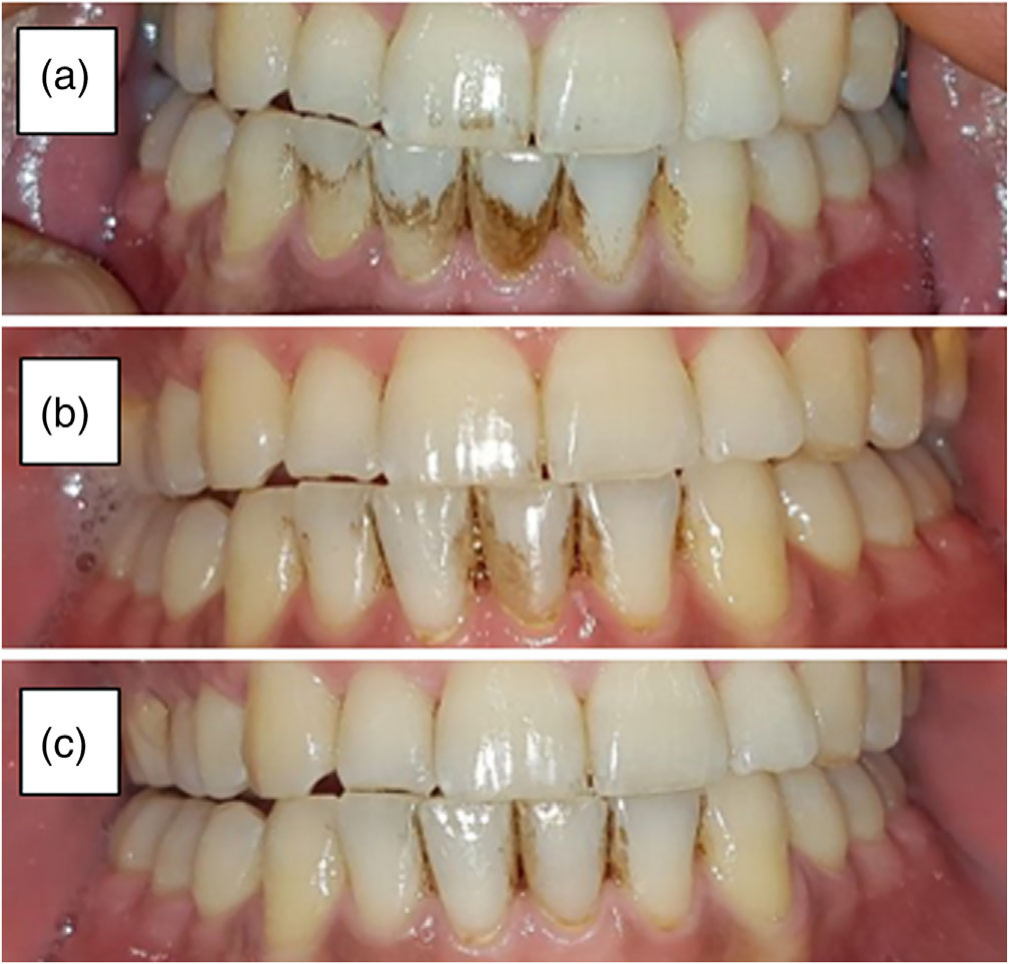
Effect on teeth stains, as illustrated by photographs of subject # 12 taken before (a), after 4 (b), and 8 (c) weeks of brushing

In addition to the whitening effect that was measured by the Vita EasyShade device, a positive effect on visible dark stains was observed, using photographs taken before and after 4 and 8 weeks of brushing. The subject photographed in Figure 4 (subject # 12) exemplifies a unique case of dark notable stains on the lower front teeth at baseline, and thus was chosen for representation of the RF effect on extrinsic dental stains. There were other subjects in the study that exhibited visible reduction in stains, however these cases were not as notable as the case of subject 12 who started at baseline with extremely dark stains that cover large portions of the lower front teeth surface. Figure 4 indicates a gradual, notable reduction of teeth stains, which were cleared almost entirely following 8 weeks of brushing.

### 3.2 Safety

Safety analysis was conducted using all recruited subjects that conducted one or more brushing sessions. No device‐related adverse events were reported during the study. There were no serious AEs, and no participants with AEs that led to discontinuation of treatment or withdrawal from the study.

## 4 DISCUSSION

Teeth whitening and stain removal can rarely be achieved by regular brushing. However, in the current study, we demonstrated that a novel RF‐based toothbrush, while used in a manual manner (no mechanical vibration), induced a statistically significant improvement in teeth shade, achieving an average reduction of 1.25 points according to the VITA standard whitening index scale. The study results also indicate that individuals with dark extrinsic teeth stains benefit from visible stain reduction, as evaluated by a visual assessment using a digital before and after photos. Following 4 and 8 weeks of brushing. The RF effect was evaluated in this study using a manual brushing mode with a standard manual brushing technique, and no mechanical vibration was activated. Nevertheless, the effect on teeth shade was found statistically significant, thus rejecting our null hypothesis that RF‐utilizing toothbrush will not significantly affect teeth shade and will not provide a notable change in extrinsic stains that are present on the tooth surfaces. The RF toothbrush does not induce teeth bleaching; during brushing with RF, the teeth shade is whitened by a thorough cleaning that exposes the natural teeth color. The measured shade whitening scores and visible stain removal represent enhanced cleaning of the teeth surface; therefore, we do not expect the whitening extent to be comparable to that of bleaching procedures, and the clinical significance of the study results should be interpreted according to this expectation.

Similar results of stains reduction and teeth whitening were achieved with a vibrating mode of the RF toothbrush (ToothWave), in a comparative single‐blind 6‐weeks study, testing the RF‐toothbrush against a regular ADA‐accepted sonic vibrating toothbrush (Milleman, Grahovac, et al., 2020; Milleman, Levi, et al., 2020)^.^ The results showed that following 6 weeks of brushing, the RF test group demonstrated statistically significant reductions in stains and discoloration compared to the control. A second comparative single‐blind 6‐weeks study tested the effect of this RF‐toothbrush (in a vibrating mode) on calculus, plaque and gingival inflammation (Milleman, Grahovac, et al., 2020). The results indicated statistically significant reductions in plaque and gingivitis in the test group as compared to the control. In addition, while the control group exhibited calculus accumulation, the test group showed a reduction in calculus over the course of the study. During these studies the RF effect combined with mechanical vibration was tested, in order to simulate the effect of the vibrating brush mode in final RF‐based product (ToothWave), and was compared to the effect of a sonic toothbrush, that utilizes mechanical vibration only without RF. These studies provide supporting evidence for the unique technological feature, which utilizes RF energy that streams on the teeth surface during brushing, and further demonstrate that the benefits obtained with the brush are attributed to the RF feature that is uniquely utilized by the RF‐based toothbrush.

There are several references in the literature to “electronic toothbrushes,” which are also referred to as “ionic toothbrushes.” These toothbrushes produce a low‐level direct electrical current that streams from the brush head into the oral cavity, using a power source (battery or solar) and a metal rod conductor (Deshmukh et al., 2006; Galgut, 1996; Hotta & Aono, 1992; Moreira et al., 2008; Perry et al., 2017; Sato et al., 2015; van der Weijden et al., 1995, 2002; van Swol et al., 1996). The scientific data available on these electronic toothbrushes is highly inconsistent. Although in vitro studies indicate their potential effect on removal of bacterial biofilms, mixed results were reported on reduction of microbial activity (Perry et al., 2017; Sato et al., 2015). Moreover, while some clinical studies indicate that a significant benefit on plaque (Deshmukh et al., 2006; Galgut, 1996) or gingival inflammation (van Swol et al., 1996) was observed, most of the clinical evidence concludes that the performance of an electronic toothbrush does not differ from that of a conventional one (Hotta & Aono, 1992; Moreira et al., 2008; van der Weijden et al., 1995, 2002), and no beneficial effect on teeth shade or stains was reported in any of the studies.

The unique beneficial effect observed in this study may be explained by technological differences between the RF‐based toothbrush and the electronic toothbrushes described in the literature. The electronic toothbrush utilizes a direct electrical current (DC), which runs from the brush into the oral cavity through the body and arm back to the brush handle (van der Weijden et al., 1995). Instead, the RF‐brush utilizes RF energy, which is an alternating electrical current (AC) that streams back and forth between two electrodes, providing a localized effect that is limited to the teeth surface. The high frequency of the alternating current that is set by the RF parameters allows to safely increase the electrical power as compared to DC current, and thus achieve more significant results (Dalziel, 1972). The difference in the effect of the RF‐based and electronic toothbrushes, results from the type of current that is utilized (alternating vs. direct) and its intensity. The RF‐brush head includes an electro‐mechanical silicon barrier, which located between the RF electrodes, and enables the observed effect by directing the current toward the teeth surface. Moreover, the RF current tends to flow along the surfaces of electrical conductors, what is known as the “skin effect” (Popovic & Popovic, 1999), and thus directing the current toward the teeth surface. Furthermore, when compared to a standard powered or manual toothbrush, the electric current is theorized to reach hard‐to‐reach areas (i.e., between the teeth) as these areas and surfaces would otherwise be chronically missed using traditional mechanical means (i.e., bristles).

Despite their technological differences, both the RF‐based and the electronic toothbrushes share the same mechanism of action, which is based on the principle of polarity that every element in nature has a positive or negative charge (Deshmukh et al., 2006). The electronic toothbrushes induce an electric charge, which is postulated to damage electrostatic bonding of plaque proteins to tooth surfaces; thus, enhancing plaque removal (Galgut, 1996). Similarly, since the RF alternating current streams close to the tooth, it brings the charged molecules that are present in the toothpaste close to the tooth surface and changes the chemical environment around it. Once these molecules accumulate near the tooth surface the chemical balance is shifted toward the removal of compounds that are electrostatically attached, replacing them by other, non‐staining charged substances, which might have greater affinity to the surface area (for instance fluoride).

We hypothesize that by changing the local charges around the tooth the alternating electrical current is able to disturb the electro‐chemical balance on the tooth surface and remove substances (i.e., stains) that are otherwise attached strongly to the enamel layer. We assume that the electrically charged toothpaste ingredients take part in the process that occurs on the teeth surface. Toothpastes are water‐based complex mixtures of abrasives and surfactants, humectants, binders, and other active ingredients. All available toothpastes contain charged molecular compounds that once the RF is activated, act as electrolytes in the medium, carry the charges along the tooth surface, and achieve the desired effect.

Notwithstanding the significant results reported herein, one of the current study limitations is its open label single arm design, which is known to be less reliable compared to a randomized controlled trial (RCT). Yet, although RCTs are the gold standard for evaluating new treatments, single‐arm studies are often utilized, when a reference to other relevant published data is available. The currently reported preliminary study results are supported by earlier publications, where the RF‐toothbrush was compared with a sonic vibrating toothbrush and was found superior in all the tested parameters (Milleman, Grahovac, et al., 2020; Milleman, Levi, et al., 2020); furthermore, an additional RCT is on‐going, aiming to compare the beneficial effect of RF‐utilizing toothbrush with those of a sonic vibrating toothbrush on stains and teeth shade. The second limitation of the current study is its small sample size, which was deemed sufficient for providing initial results within the scope of a pilot study.

## 5 CONCLUSIONS

Within the limit of the present study, the RF‐based toothbrush was shown to produce a significant reduction in extrinsic dental stains and to achieve a significant improvement in whitening of the teeth shade following 4 and 8 weeks of brushing. These benefits were also confirmed in a randomized clinical study, comparing the Toothwave to a sonic vibrating toothbrush and thus are attributed to the RF feature that is uniquely utilized by the RF‐based toothbrush.

## AUTHOR CONTRIBUTIONS

**Shadi Shehadeh:** Performed the screening and clinical observations during baseline and follow up visits. **Liora Levi:** Responsible for study monitoring on behalf of the sponsor. **Dror Shamir:** Principle investigator of the study.

## CONFLICT OF INTEREST

This study was funded by Home Skinovations LTD, of whom Liora Levi is an employee.

## ETHICS STATEMENT

The study protocol was approved by ethics committee Helsinki at the Ministry of Health in Israel (No. 0073‐16‐BNZ). All the participants gave written informed consent for inclusion in this study.

## Data Availability

The data that support the findings of this study are available from the corresponding author upon reasonable request.

## References

[cre2460-bib-0001] Belenky, I. , Margulis, A. , Elman, M. , Bar‐Yosef, U. , & Paun, S. D. (2012). Exploring channeling optimized radiofrequency energy: A review of radiofrequency history and applications in esthetic fields. Advances in Therapy, 29(3), 249–266. 22382873 10.1007/s12325-012-0004-1

[cre2460-bib-0002] Clifton, M. C. (2014). Tooth whitening: What we now know. The Journal of Evidence‐Based Dental Practice, 14, 70–76. 10.1016/j.jebdp.2014.02.006 PMC4058574 24929591

[cre2460-bib-0003] Dahl, J. E. , & Pallesen, U. (2003). Tooth bleaching‐a critical review of the biological aspects. Critical Reviews in Oral Biology and Medicine, 14(4), 292–304. 12907697 10.1177/154411130301400406

[cre2460-bib-0004] Dalziel, C. F. (1972). Electric shock hazard. IEEE Spectrum, 9(2), 41–50.

[cre2460-bib-0005] Deshmukh, J. , Vandana, K. L. , Chandrashekar, K. T. , & Savitha, B. (2006). Clinical evaluation of an ionic tooth brush on oral hygiene status, gingival status, and microbial parameter. Indian Journal of Dental Research, 17(2), 74–77. 17051872 10.4103/0970-9290.29887

[cre2460-bib-0006] Galgut, P. N. (1996). Efficacy of a new electronic toothbrush in removing bacterial dental plaque in young adults. General Dentistry, 44(5), 441–445. 9171045

[cre2460-bib-0007] Goldberg, M. , Grootveld, M. , & Lynch, E. (2010). Undesirable and adverse effects of tooth‐whitening products: A review. Clinical Oral Investigations, 14(1), 1–10. 19543926 10.1007/s00784-009-0302-4

[cre2460-bib-0008] Greenwall, L. (2008). The dangers of chlorine dioxide tooth bleaching. Aesthetic Dentistry Today, 2, 20–22.

[cre2460-bib-0009] Hara, A. T. , & Turssi, C. P. (2017). Baking soda as an abrasive in toothpastes: Mechanism of action and safety and effectiveness considerations. Journal of the American Dental Association (1939), 148(11S), S27–S33. 29056187 10.1016/j.adaj.2017.09.007

[cre2460-bib-0010] Hotta, M. , & Aono, M. (1992). A clinical study on the control of dental plaque using an electronic toothbrush with piezo‐electric element. Clinical Preventive Dentistry, 14(4), 16–18. 1521397

[cre2460-bib-0011] Mahaseth, A. , & Kuzminov, T. (2017). Potentiation of hydrogen peroxide toxicity: From catalase inhibition to stable DNA‐iron complexes. Mutation Research, 773, 274–281. 28927535 10.1016/j.mrrev.2016.08.006 PMC5607474

[cre2460-bib-0012] Milleman, K. R. , Grahovac, T. L. , Yoder, A. L. , Levi, L. , & Milleman, J. L. (2020). Safety and efficacy of a novel toothbrush utilizing RF energy for teeth shade whitening and the reduction of teeth stains. Advances in Dentistry & Oral Health, 12(3), 214–220. 32470241

[cre2460-bib-0013] Milleman, K. R. , Levi, L. , Grahovac, T. L. , & Milleman, J. L. (2020). Safety and efficacy of a novel toothbrush utilizing RF energy for the reduction of plaque, calculus and gingivitis. American Journal of Dentistry, 33, 151–156. 32470241

[cre2460-bib-0014] Minoux, M. , & Serfaty, R. (2008). Vital tooth bleaching: Biologic adverse effects‐a review. Quintessence International, 39(8), 645–659. 19107251

[cre2460-bib-0015] Moreira, C. H. C. , Luz, P. B. , Villarinho, E. A. , Petri, L. C. , & Rösing, C. K. (2008). Efficacy of an ionic toothbrush on gingival crevicular fluid—A pilot study. Acta Odontológica Latinoamericana, 21(1), 17–20. 18841741

[cre2460-bib-0016] Perry, C. N. , Beard, R. D. , Lolley, R. J. , Saunders, L. E. B. , Quest, D. , & O'Donnell, J. (2017). Energy output and in vitro biologic effects of an ionic toothbrush. Texas Dental Journal, 134(4), 236–245. 30725552

[cre2460-bib-0017] Popovic, Z. , & Popovic, B. (1999). The skin effect: Introductory electromagnetics. Prentice‐Hall.

[cre2460-bib-0018] Reis, A. , Tay, L. Y. , & Herrera, D. R. (2011). Clinical effects of prolonged application time of an in‐office gel bleaching gel. Operative Dentistry, 36(6), 590–596. 21913864 10.2341/10-173-C

[cre2460-bib-0019] Sato, T. , Hirai, N. , Oishi, Y. , Uswak, G. , Komiyama, K. , & Hamada, N. (2015). Efficacy of a solar‐powered TiO_2_ semiconductor electric toothbrush on P. gingivalis biofilm. American Journal of Dentistry, 28(2), 81–84. 26087572

[cre2460-bib-0020] Schallreuter, K. U. , & Elwary, S. (2007). Hydrogen peroxide regulates the cholinergic signal in a concentration dependent manner. Life Sciences, 80(24–25), 2221–2226. 17335854 10.1016/j.lfs.2007.01.028

[cre2460-bib-0021] Sulieman, M. A. (2008). An overview of tooth‐bleaching techniques: Chemistry, safety and efficacy. Periodontology 2000, 48, 148–169. 18715362 10.1111/j.1600-0757.2008.00258.x

[cre2460-bib-0022] van der Weijden, G. A. , Timmerman, M. F. , Piscaer, M. , Snoek, I. , van der Velden, U. , & Galgut, P. N. (2002). Effectiveness of an electrically active brush in the removal of overnight plaque and treatment of gingivitis. Journal of Clinical Periodontology, 29(8), 699–704. 12390566 10.1034/j.1600-051x.2002.290806.x

[cre2460-bib-0023] van der Weijden, G. A. , Timmerman, M. F. , Reijerse, E. , Mantel, M. S. , & Van der Velden, U. (1995). The effectiveness of an electronic toothbrush in the removal of established plaque and treatment of gingivitis. Journal of Clinical Periodontology, 22(2), 179–182. 7775675 10.1111/j.1600-051x.1995.tb00130.x

[cre2460-bib-0024] van Swol, R. L. , van Scotter, D. E. , Pucher, J. J. , & Dentino, A. R. (1996). Clinical evaluation of an ionic toothbrush in the removal of established plaque and reduction of gingivitis. Quintessence International, 27(6), 389–394. 8941832

